# A multi-category eye image dataset for AI-based segmentation and analysis of eyelid disorders

**DOI:** 10.1038/s41597-026-08179-y

**Published:** 2026-08-29

**Authors:** Ji Shao, Jing Cao, Changjun Wang, Peifang Xu, Xuan Zhang, Yiming Sun, Pengjie Chen, Ningxin Dai, Lixia Lou, Juan Ye

**Affiliations:** Zhejiang University, Eye Center of Second Affiliated Hospital, School of Medicine, China. Zhejiang Provincial Key Laboratory of Ophthalmology. Zhejiang Provincial Clinical Research Center for Eye Diseases. Zhejiang Provincial Engineering Institute on Eye Diseases, Hangzhou, China

**Keywords:** Eyelid diseases, Eye manifestations

## Abstract

Abnormal eyelid position and morphology can cause visual dysfunction, ocular surface damage, and facial deformities, highlighting the need for accurate eyelid assessment. Advances in artificial intelligence (AI) have enabled automated analysis of eyelid abnormalities using external eye images, but development is limited by the lack of publicly available datasets with multi-category disorders and standardized structural annotations. To address this gap, we constructed a clinically annotated eye image dataset of 1,414 images from eight common eyelid disorders and a normal control group. Each image includes diagnostic labels and manual annotations of three key periocular structures: eyelid fissure, cornea, and eyebrow. Image quality evaluation and expert verification were performed to ensure annotation reliability. Inter- and intra-annotator consistency demonstrated excellent agreement. A baseline Attention 2D U-Net segmentation model trained on the dataset achieved Dice coefficients of 0.93, 0.96, and 0.89 for the eyelid fissure, cornea, and eyebrow, respectively. This dataset provides a valuable resource for automated segmentation, quantitative periocular measurement, and AI-assisted eyelid analysis, supporting standardized and reproducible approaches for eyelid assessment.

## Background & Summary

The normal position and morphology of the eyelids are crucial to maintain proper vision function and appearance. Due to the close anatomical relationship between the eyelid and ocular surface, the morphological abnormalities of the eyelids may lead to vision impairment, ocular surface damage, exposure keratitis, and even loss of vision^1–3^. In addition to their functional role, the eyelids and surrounding periocular region are critical determinants of facial aesthetics. As such, eyelid disorders may adversely affect psychological development in children and contribute to appearance-related anxiety and reduced quality of life in adults^4,5^. Early detection and accurate assessment of eyelid abnormalities are therefore important for both clinical management and long-term patient outcomes.

Precise measurements of the eyelid position and contour are the key points of clinical decision-making and treatment planning in various eyelid disorders, as they guide interventions such as surgical corrections, severity assessment, disease monitoring, and treatment evaluation^6–8^. Traditionally, these measurements are obtained manually using a ruler. However, manual assessment is time-consuming, requires substantial clinical experience and patient cooperation, and is susceptible to measurement variability^9^. Furthermore, conventional measurements typically focus on a limited number of parameters, such as eyelid height, while providing only limited information regarding overall periocular morphology. These limitations restrict the efficiency, reproducibility, and scalability of eyelid assessment in modern clinical practice.

With the widespread availability of digital imaging and advances in artificial intelligence (AI), recent studies have demonstrated the potential of using external eye photographs for automated diagnosis and analysis of eyelid abnormalities^10–17^. However, these studies have generally relied on relatively small datasets, and the underlying annotations are often not publicly available, limiting reproducibility and wider adoption^18–20^. Several publicly accessible eye image datasets, such as TEyeD^21^ and OpenEDS^22^, primarily contain images from healthy subjects and are not specifically designed for clinical eyelid disorder analysis. Consequently, there remains a lack of publicly available, clinically annotated, multi-category datasets with standardized structural annotations of key periocular structures.

To address this gap, we constructed a clinically annotated multi-category eye image dataset containing 1,414 images from eight common eyelid disorders and a normal control group. Each image is accompanied by diagnostic labels and precise manual annotations of three clinically important periocular structures: the eyelid fissure, cornea, and eyebrow. Inter- and intra-annotator consistency analyses demonstrated excellent annotation reliability, and baseline Attention 2D U-Net segmentation experiments further validated the utility of the dataset for automated analysis.

The main contributions of this dataset are threefold:

i) Comprehensive multi-category coverage: Includes eight common eyelid disorders and normal eyes, supporting AI research across diverse clinical scenarios.
ii) High-quality expert annotations: Manual segmentations of the eyelid fissure, cornea, and eyebrow with validated inter- and intra-annotator consistency, enabling quantitative periocular analysis.
iii) Demonstrated utility: Baseline Attention 2D U-Net experiments demonstrate the dataset’s applicability for automated segmentation tasks.

Collectively, this dataset provides a valuable resource for automated segmentation and objective periocular measurement, supporting future research on AI-assisted analysis of eyelid disorders and standardized eyelid assessment.

**Fig. 1:**
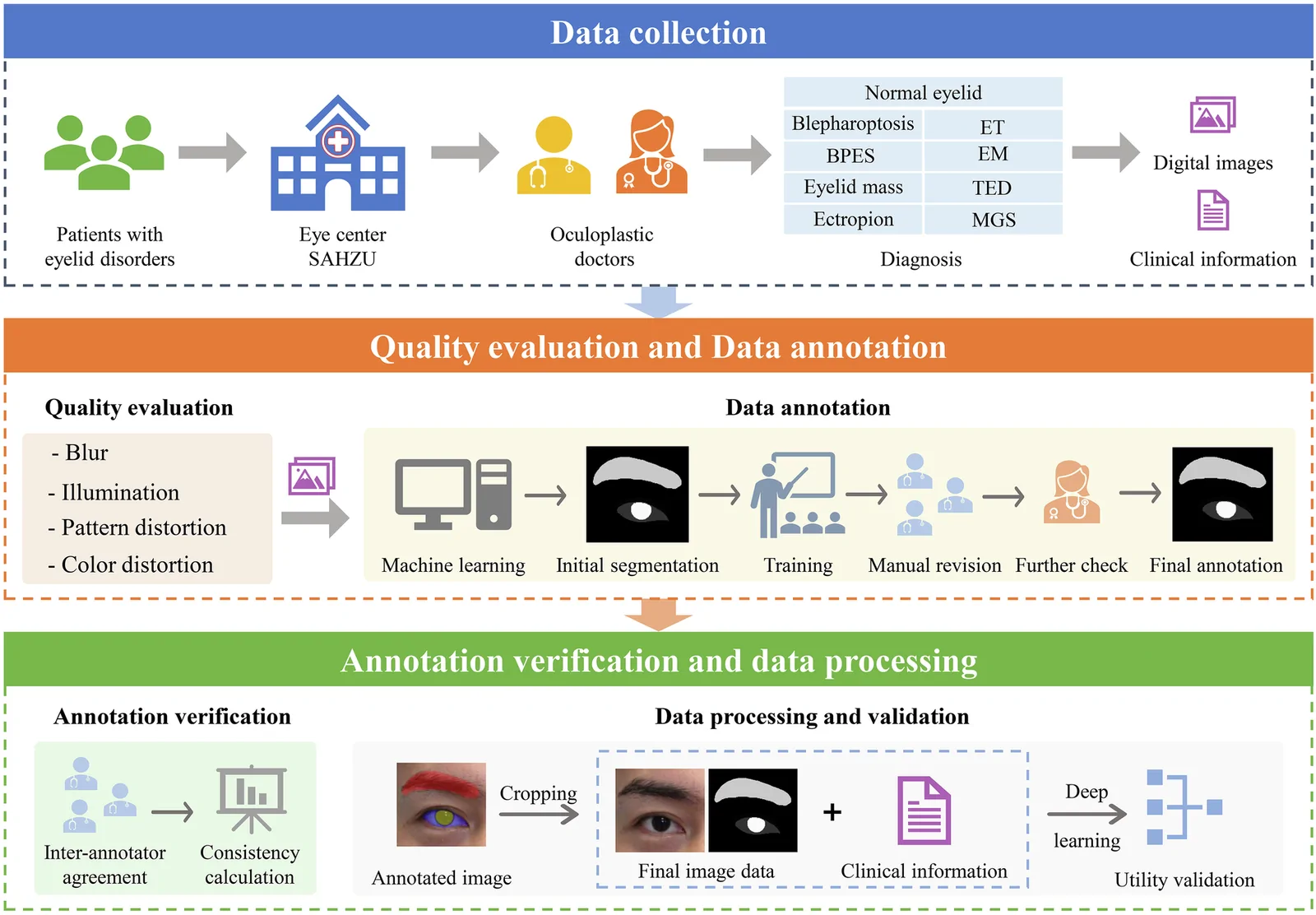
The workflow of this work. Our work comprised 3 main steps. First, oculoplastic specialists conducted thorough clinical assessments and made diagnoses. Image data that met the inclusion criteria were collected. In the second step, a quality assessment was performed to retain high-quality images. Preliminary annotations were generated by a machine learning model and subsequently refined by a trained annotator team to ensure accurate labeling of eyelid structures. In the final step, intra- and inter-annotator agreement were verified. The eyelid regions were cropped and paired with corresponding masked images for subsequent validation of artificial intelligence-based analysis. BEPS, Blepharophimosis-plosis-epicanthus inverse syndrome; ET, Entropion and trichiasis; EM, eyelid malformation; TED, thyroid eye disease; MGS, Marcus-Gunn syndrome.

Figure 1 illustrates the overall workflow of the study, including data collection, quality control, annotation, validation, processing, and dataset construction.

## Methods

### Ethics declaration

This study followed the principles of the Declaration of Helsinki. Approval for this study was obtained from the Ethic Committee of the Second Affiliated Hospital of Zhejiang University, School of Medicine (No. 2020-583). This study was registered on ClinicalTrials.gov with trial registration number NCT04921020. Written informed consent complying with the requirements of the Medical Ethics Committee was signed by every adult participant and the guardian of minors.

### Patient selection and data collection

All participants included in this study attended Zhejiang University, Eye Center of Second Affiliated Hospital, School of Medicine, from 2020 to 2024. The types of eyelid diseases included in this study and the corresponding diagnosis criteria were as follows: (1) blepharoptosis was diagnosed if upper eyelid margin covered corneal upper margin more than 2 mm when gazing at the primary position, and was classified into the mild, moderate and severe group according to the function of levator muscle^23^; (2) ectropion was diagnosed if lower eyelid was pulled outward away from the eyeball resulting in the exposure of the lower eyelid conjunctiva^24^; (3) entropion and trichiasis was diagnosed as inward rotation of eyelid and resulted the contact between eyelashes and cornea^24,25^; (4) thyroid eye disease (TED) was diagnosed according to Bartley criteria^26^; (5) blepharophimosis-plosis-epicanthus inverse syndrome was diagnosed according to the complex eyelid malformation present at birth, which was characterized by blepharophimosis, ptosis, epicanthus inversus, and telecanthus^27^; (6) Marcus-Gunn syndrome was diagnosed as congenital blepharoptosis with ptotic eyelid elevating when chewing^28^; (7) eyelid malformation was defined as morphological abnormalities of the eyelid secondary to ocular surgery or trauma; (8) eyelid mass was diagnosed according to the pathological results of excision; Normal eyelid was defined as having no morphological or structural abnormalities, except for aging-related changes.

The diagnosis of each participant was given by the first-contact oculoplastic surgeon and was confirmed by an oculoplastic expert with more than 20 years of experience. Exclusion criteria were as follows: (1) uncooperative patients; (2) patients with iris or sclera abnormalities; (3) coexisting systemic disease affecting eyelid appearance.

All digital images were taken using a Canon or Sony digital camera (Canon EOS 1500 D, Canon Corporation, Japan; Sony ILCE-7RM4A, Sony, Japan) with participants gazing at the primary position. The camera was fixed 50 to 100 mm in front of the participant at eye level in a room with the same light conditions. Basic information was recorded, including gender, age, and laterality.

### Data annotation

This study used a two-step annotation method, which included machine segmentation and manual annotation as the main processes. This human-computer collaborative annotation method helped clinicians save time.

In the machine learning segmentation process, digital images were put into recurrent residual convolutional neural networks based on U-Net for the initial segmentation of the cornea, eyelid fissure, and eyebrow. The segmented mask was recorded in the RGB mode with the red, green, and blue channels being all set as 200, 250, and 50 for the eyebrow, cornea, and eyelid fissure region, respectively. In the manual annotation process, an annotator group consisting of 3 ophthalmology residents and 1 oculoplastic expert was first formed. A detailed annotation guideline was prepared by the oculoplastic expert. After learning this guideline, each resident was assigned a total of 45 images, with 5 randomly selected from each of the 9 eyelid subsets. The manually annotated images were reviewed by the expert, and the annotations were sent back to the resident for revision if they did not meet the standard. The manual process officially began when the annotations from all 3 residents met the required standards. The dataset was divided into 3 subsets, and each resident was asked to correct 1 subset on the corresponding initial segmentation of the cornea, eyelid fissure, and eyebrow using Photoshop software (Photoshop CC, Adobe Systems, USA). The oculoplastic expert further revised the annotations if there were any unclear annotation results. Final annotations were completed after being checked by the expert.

### Image processing

Image processing consisted of two main steps: image quality assessment before annotation and region extraction after annotation.

High-quality images are essential for accurate annotation and subsequent quantitative analysis. Therefore, all images underwent a quality assessment procedure before annotation. Four aspects were evaluated, including image blurring, illumination, pattern distortion, and color distortion. Two senior ophthalmologists independently reviewed each image and assigned a binary score (0 = unacceptable, 1 = acceptable) for each criterion. Subsequently, an oculoplastic specialist performed a final review. Images that failed any of the quality criteria were excluded from the dataset.

After annotation, the periocular region corresponding to a single eye was extracted from each image. Cropping boundaries were determined based on the annotated eyelid fissure, cornea, and eyebrow, with a consistent extension applied beyond the annotated structures to ensure complete coverage of the region of interest. Corresponding annotation masks were cropped simultaneously to preserve spatial alignment with the original image. The cropped images retained their original resolution, and no additional image resizing, enhancement, or color correction procedures were performed.

In this dataset, only one eye per patient was included. For unilateral cases, the affected eye was selected. For bilateral cases, the eye exhibiting more prominent clinical manifestations was included. In the normal eyelid subset, either the left or right eye was randomly selected to ensure consistency in data representation.

## Data Records

The proposed eyelid dataset has been uploaded to Synapse in zip format under a Data Usage Agreement (DUA)^29^. Table 1 exhibited the composition and distribution of this dataset, which included 8 different subsets of eyelid disorders and a cross-age normal eyelid subset from 1414 eye images of 1399 individuals. The unzipped file contains 3 parts: the eye images, the annotation images, and the summary lists.

**Table 1:** The composition and distribution of this dataset.

	Number of participants		Number of eyes	
**Blepharoptosis**	mild	55	mild	55
	moderate	183	moderate	183
	severe	124	severe	124
**Ectropion**	12		12	
**Entropion and trichiasis**	upper eyelid	20	upper eyelid	20
	lower eyelid	251	lower eyelid	251
	upper and lower eyelid	60	upper and lower eyelid	60
**TED**	81		81	
**BPES**	9		9	
**MGS**	15		30	
**EM**	13		13	
**Eyelid mass**	upper eyelid	49	upper eyelid	49
	lower eyelid	71	lower eyelid	71
	canthal mass	2	canthal mass	2
**Normal eyelid**	454		454	
**Total**	1399		1414	

TED, thyroid eye disease; BPES, blepharophimosis-plosis-epicanthus inverse syndrome; MGS, Marcus-Gunn syndrome; EM, eyelid malformation;

Each image file consists of 9 folders, named “Blepharoptosis”, “Ectropion”, “Entropion and trichiasis”, “Thyroid eye disease”, “Blepharophimosis-plosis-epicanthus inverse syndrome”, “Marcus-Gunn syndrome”, “Eyelid malformation”, “Eyelid mass”, and “Normal eyelid”, respectively. Among these folders, the “Entropion and trichiasis” folder and the “Eyelid mass” folder are divided into 3 subfolders, according to the location of lesions. The “Blepharoptosis” folder is separated into mild, moderate, and severe subsets according to different levator functions. All images are saved in a PNG format in the eyelid image dataset with the name “N_eye/mask _laterality.png”, and the corresponding annotations are stored in the annotation folders with the same name and image format. Except for the “Marcus-Gunn syndrome” subset, each image in the other 8 subsets corresponds to a single participant. In the “Marcus-Gunn syndrome” subset, since the upper eyelid of a patient usually has synkinetic movements during jawing, two images from each patient were included. The first image taken with their mouth closing was named “Na_eye/mask_laterality.png”, while the second image taken at the moment of their mouth opening was named “Nb_eye/mask_laterality.png”. N represents the order in the subfolder.

The summary list shows the number, laterality, age group, and gender of the recruited participants, respectively. To convert the pixel values of the image into real-world distances, the scale ratio between millimeters and pixels is also provided in the summary list. In the TED, blepharoptosis, blepharophimosis-plosis-epicanthus inverse syndrome, ectropion, Marcus-Gunn syndrome, and eyelid malformation subsets, a circular marker with a diameter of 10 millimeters was affixed to each patient’s forehead. The scale ratio was subsequently determined by dividing the marker’s actual diameter by its pixel diameter in the image. In the other subsets, the scale transformation was still based on this marker for patients younger than 4 years. For those minors aged 4 years and older, the corneal diameter was used instead, as it remains stable and age-independent beyond this point^30^. Similarly, for adult patients, the white-to-white distance was applied as the horizontal corneal measurement^31^. In the entropion and trichiasis, and normal eyelid subsets, the average corneal diameter of both eyes was used for transformation. In contrast, for the eyelid mass subset, the corneal diameter of the healthy fellow eye was adopted.

## Technical Validation

### Annotation evaluation

Considering the importance of annotation stability for the usage of this dataset, the evaluation of intra- and inter-annotators was carried out. A total of 30 images were randomly selected from the dataset. To assess the inter-annotator consistency, three ophthalmology residents (R1, R2, and R3) manually segmented these images. Dice coefficient and Intersection over Union (IoU) were measured to make comparisons between different annotators (R1 vs R2; R1 vs R3; R2 vs R3). To assess the intra-annotator consistency, resident R1 was asked to generate the manual annotation on the same randomly selected dataset with a 30-day gap. Then, the Dice coefficient and IoU were calculated between the 1st and 2nd annotations.

**Fig. 2:**
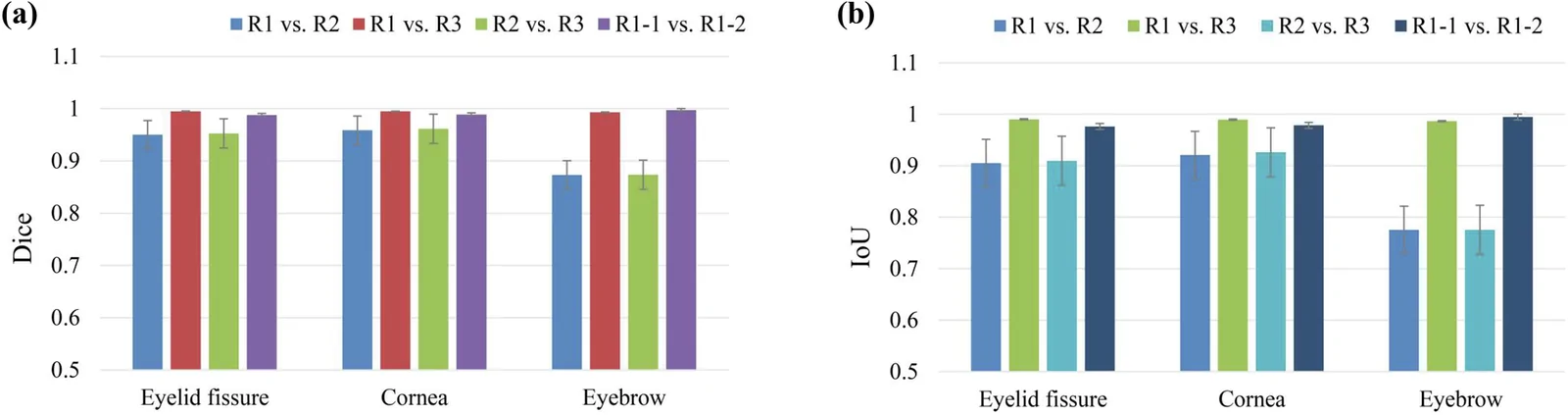
The intra- and inter-annotator agreement was verified in this dataset. (**a**) The Dice coefficient of annotated eyelid fissure, cornea, and eyebrow. (**b**) The IoU of annotated eyelid fissure, cornea, and eyebrow. R, resident.

Figure 2 shows the Dice coefficient and IoU of the eyelid fissure, cornea, and eyebrow that were annotated by different annotators. The annotations of the eyelid and cornea demonstrated excellent consistency, with all Dice coefficients and IoU exceeding 0.90 between any 2 annotators. The eyebrow area is challenging to annotate due to its sparse structure and indistinct boundaries. However, our study achieved satisfactory annotation results, with a Dice coefficient exceeding 0.87 and an IoU greater than 0.77. For intra-annotator consistency of Resident 1, the Dice coefficient of the annotated eyelid, cornea, and eyebrow surpassed 0.98, while IoU exceeded 0.97, indicating excellent stability and minimal intra-annotator error.

### Dataset utility

The proposed dataset contains various eyelid morphological disorders and expert-reviewed annotations of the eyelid fissure, cornea, and eyebrow, enabling the utility of automatic segmentation and precise ocular measurement based on deep learning. To demonstrate the utility of the proposed dataset for AI-based image analysis, a baseline semantic segmentation experiment was conducted using an Attention 2D U-Net architecture. All images with corresponding annotations of the eyelid fissure, cornea, and eyebrow were included in the experiment. The dataset was randomly divided into training, validation, and testing subsets at a ratio of approximately 8:1:1. Dice coefficient and IoU were used to evaluate segmentation performance.

The model achieved Dice coefficients of 0.93, 0.96, and 0.89 for the eyelid fissure, cornea, and eyebrow, respectively, with corresponding IoU values of 0.88, 0.92, and 0.80 (Table 2). Among the three anatomical structures, the cornea achieved the highest segmentation performance owing to its relatively clear boundary and consistent morphology. The eyelid fissure also demonstrated strong segmentation performance despite substantial morphological variations across different eyelid disorders. Although the eyebrow region exhibited relatively lower performance due to its sparse structure and less distinct boundaries, the results remained satisfactory and demonstrated the feasibility of automatic eyebrow segmentation. These findings provide objective evidence that the proposed dataset contains high-quality annotations and can effectively support the development of deep learning–based segmentation algorithms for periocular image analysis.

**Table 2:** Baseline image segmentation performance.

Structure	Dice coefficient	IoU
**Cornea**	0.96	0.92
**Eyelid fissure**	0.93	0.88
**Eyebrow**	0.89	0.80

IoU, Intersection over Union.

Due to the potential sensitivity of eye images, this dataset is provided under a DUA through the Synapse platform. Researchers interested in accessing the dataset should complete the following steps. First, register for a certified Synapse account (https://www.synapse.org). Second, complete and sign the DUA and send its copy along with Synapse account information to the corresponding author, copying shaoji@zju.edu.cn (data access contact). Data access will be provided to all users that agree to the terms and conditions of the DUA. This multi-category eye image dataset offers origin images of 8 common eyelid disorders and 1 normal control with standardized annotations of the cornea, eyelid fissure, and eyebrow. The entire dataset could be downloaded from the link^29^. Researchers using this dataset should cite this Data Descriptor and acknowledge the dataset in any derived publications. All data use must comply with the signed DUA. The agreement is available in the Supplementary Information.

## Supplementary information

Supplemental

## Data Availability

The dataset described in this study is available through Synapse^29^ under a Data Usage Agreement (DUA). The repository contains the eye images, corresponding annotations, and summary files described in the Data Records section. Access will be provided upon approval of the DUA. The dataset is available at https://www.synapse.org/Synapse:syn74332552.

## Code availability

No custom code was used in the construction of the dataset.
