# Supplementary material for: A multi-category eye image dataset for AI-based segmentation and analysis of eyelid disorders
Source: Sci Data. 2026 Aug 29;13:1281. doi: 10.1038/s41597-026-08179-y (PMC13545276; doi:10.1038/s41597-026-08179-y)
Supplement: Supplementary file 1 [file 41597_2026_8179_MOESM1_ESM.docx]

**Supplemental**

**A multi-category eye image dataset for AI-based segmentation and analysis of eyelid disorders**

**Data acquisition process**

1. Register for a Synapse account.

2. Become a Synapse Certified User with a validated user profile.
3. Contact the corresponding author and submit a signed Data Usage Agreement, together with the user's Synapse account information, copying shaoji@zju.edu.cn (data access contact).
4. Obtain permission to download the data.

**Data Usage Agreement**

The data may be used only if they are used and protected in accordance with the terms and conditions stated in this License. It is hereby agreed between the data requestor, hereinafter referred to as the “LICENSEE”, that:

1. The LICENSEE will not attempt to identify any individual or institution referenced in the restricted data from the multi-category eye image dataset.
2. The LICENSEE will exercise all reasonable and prudent care to avoid disclosure of the identity of any individual or institution referenced in the restricted data from the multi-category eye image dataset in any publication or other communication.
3. The LICENSEE will not share access to the restricted data from the multi-category eye image dataset with any third party.
4. The LICENSEE will exercise all reasonable and prudent care to maintain the physical and electronic security of the restricted data from the multi-category eye image dataset.
5. If the LICENSEE finds information within the restricted data from the multi-category eye image dataset that he or she believes might permit identification of any individual or institution, the LICENSEE will report the location of this information promptly by email to [**yejuan@zju.edu.cn**](mailto:yejuan@zju.edu.cn), citing the location of the specific information in question.
6. The LICENSEE will use the data for the sole purpose of lawful use in scientific research and no other.
7. The LICENSEE agrees to contribute code associated with publications arising from this data to a repository that is open to the research community.
8. This agreement may be terminated by either party at any time, but the LICENSEE's obligations with respect to the multi-category eye image dataset shall continue after termination.

THE DATA ARE PROVIDED “AS IS”, WITHOUT WARRANTY OF ANY KIND, EXPRESS OR IMPLIED, INCLUDING BUT NOT LIMITED TO THE WARRANTIES OF MERCHANTABILITY, FITNESS FOR A PARTICULAR PURPOSE AND NONINFRINGEMENT. IN NO EVENT SHALL THE AUTHORS OR COPYRIGHT HOLDERS BE LIABLE FOR ANY CLAIM, DAMAGES OR OTHER LIABILITY, WHETHER IN AN ACTION OF CONTRACT, TORT OR OTHERWISE, ARISING FROM, OUT OF OR IN CONNECTION WITH THE DATA OR THE USE OR OTHER DEALINGS IN THE DATA.
